# A Novel Polysaccharide From *Heimioporus retisporus* Displays Hypoglycemic Activity in a Diabetic Mouse Model

**DOI:** 10.3389/fnut.2022.964948

**Published:** 2022-07-11

**Authors:** Xiaobin Feng, Peng Wang, Yuxiao Lu, Zejun Zhang, Chunxin Yao, Guoting Tian, Qinghong Liu

**Affiliations:** ^1^Department of Vegetables, College of Horticulture, China Agricultural University, Beijing, China; ^2^Department of Environment and Chemical Engineering, Tangshan College, Tangshan, China; ^3^College of Food Science and Nutritional Engineering, China Agricultural University, Beijing, China; ^4^Institute of Biotechnology and Germplasm Resources, Yunnan Academy of Agricultural Sciences, Kunming, China

**Keywords:** *Heimioporus retisporus*, polysaccharide, characterization, hypoglycemia, cardioprotective

## Abstract

A novel polysaccharide, *Heimioporus retisporus* Polysaccharide (HRP) was extracted from the edible mushroom *Heimioporus retisporus*. HRP had weight-average molecular weight 1,949 kDa and number-average molecular weight 873 kDa, and its major components were arabinose (0.71%), galactose (12.93%), glucose (49.00%), xylose (8.59%), mannose (17.78%), and glucuronic acid (10.99%). Fourier transform infrared spectroscopy and nuclear magnetic resonance spectroscopy revealed that HRP was composed of 1,3-linked β-D-glucose, 1,6-linked β-D-mannose, 1,6-linked β-D-galactose, 1,4-linked β-D-galactose, 1,4-linked β-D-xylose, and 1,5-linked α-L-arabinose. Thermogravimetric analysis indicated that degradation temperature (T_0_) of HRP was 200°C. In an STZ-induced diabetic mouse model, oral administration of HRP (40 mg/kg/d) for 28 days significantly reduced blood glucose levels, and reduced heart organ index by decreasing expression of IL-6 and TNF-α. Our findings indicate hypoglycemic effect of HRP, and its potential application as a hypoglycemic agent.

## Key Points

1. A polysaccharide HRP was purified from fruiting bodies of *Heimioporus retisporus*.
2. Preliminary structural characterization of HRP was performed.
3. Hypoglycemic activity of HRP was evaluated in a STZ-induced diabetic mouse model.

## Introduction

Diabetes is a common metabolic disorder characterized by high blood glucose level resulting from β-cell dysfunction and insulin resistance (1). It may cause damage to various organs (particularly liver, kidney, and brain), and presents increased risk of cardiovascular disease, kidney disease, and partial or complete blindness (2). It is a pro-inflammatory state associated with increased production of reactive oxygen species (ROS) and expression of inflammatory cytokines (e.g., IL-1β, IL-6, IL-8, TNF-α) that promote apoptosis (3–5). The International Diabetes Federation (IDF) estimates that ∼425 million adults worldwide have diabetes, and that this number will increase to ∼630 million by 2045.

Commonly used diabetes medications have several adverse effects, including hypoglycemia (sulfonylureas) (6), liver damage, cardiovascular disease (thiazolidinedione) (7, 8), and gastrointestinal disorders (flatulence, diarrhea, abdominal pain, nausea, vomiting) (α-glucosidase inhibitors, biguanide) (9, 10). There is an urgent need for effective diabetes medications without such adverse effects. Polysaccharides are naturally occurring compounds present in a wide variety of animals, plants, algae, microorganisms, and fungi, notably medicinal mushroom species. Numerous studies have documented beneficial biological activities of polysaccharides, including hypoglycemic, antioxidant, anticoagulant, antitumor, antimutagenic, anticomplementary, antiviral, and anti-inflammatory activities (11–13, 14). A *Hericium erinaceus* polysaccharide reduced glucose levels in normal and alloxan-induced diabetic mice without adverse effects (15), and the polysaccharide from *Ganoderma lucidum* and *Hohenbuehelia serotina* displayed hypoglycemic activity (16, 17). In many cases, activities of polysaccharides are related to their structure. Lentinan (a *Lentinula edodes* polysaccharide), for example, displayed immunomodulatory and antitumor effects, based on its β-D-glucan structure (18). However, few studies have addressed mechanisms of hypoglycemic activity as related to structure of specific polysaccharides.

*Heimioporus retisporus* is an edible mushroom (class Agaricomycetes, family Boletaceae) native to Yunnan Province (China). We previously described inhibition of endogenous oxidative stress and moisturizing effects of crude polysaccharides from *H. retisporus* (19). In the present study, as part of an ongoing search for safe, natural, hypoglycemic agents, we purified a water-soluble polysaccharide, *Heimioporus retisporus* Polysaccharide (HRP) from *H. retisporus*, characterized its chemical structure, assayed its hypoglycemic activity, and examined relationships between its structure and bioactivities.

## Materials and Methods

### Materials and Chemicals

*Heimioporus retisporus* fruiting bodies were purchased from Kunming, Yunnan Province. Ion exchange resins CM-Sepharose and DEAE-Sepharose were from General Electric Co. (United States). Metformin hydrochloride (MET) was from Beijing Coway Pharmaceutical Co. (China). L-arabinose, D-glucose, D-galactose, D-mannose, D-xylose, glucuronic acid, and galacturonic acid were from Dionex Ltd. (China), Streptozotocin (STZ) and TRIzol reagent were from Sigma-Aldrich (United States). Reverse transcription kit and polymerase chain reaction mix were from Mei5 Biotechnology Co. (Beijing, China). All other reagents used in this study were analytical grade.

### Extraction and Purification of *Heimioporus retisporus* Polysaccharide

**FIGURE 1:**
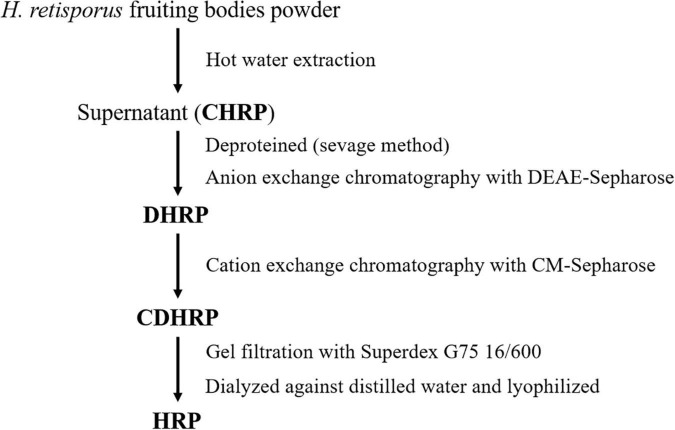
Purification of *Heimioporus retisporus* polysaccharide (HRP) (schematic).

*Heimioporus retisporus* Polysaccharide was extracted and purified as shown schematically in Figure 1. Fruiting bodies were dried at 45°C to constant weight, and ground into powder (mesh #40; particle size ∼420 μm) with a triturator. The powder was evenly dispersed in water at ratio 1:15 (w/v), the mixture was heated in a water bath 4 h at 95°C and centrifuged (10,000 rpm, 10 min), and supernatant was collected. Supernatant was mixed with ethanol at ratio 1:3 (v/v), mixture was kept 10 h at 4°C and centrifuged (10,000 rpm, 10 min), and sediment was collected as crude HRP (CHRP).

**FIGURE 2:**
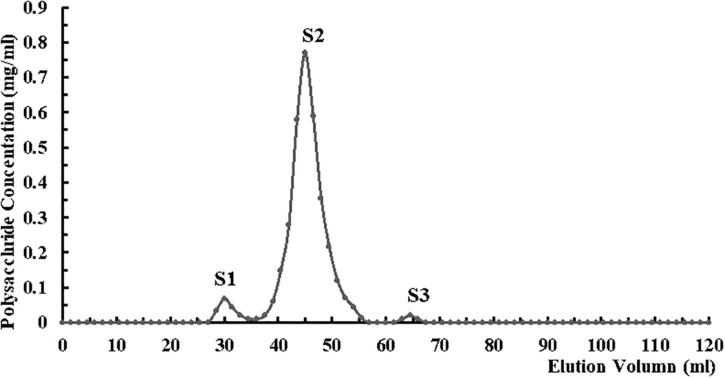
Purification of *Heimioporus retisporus* Polysaccharide (HRP) on Superdex 75 16/600 gel filtration column by fast protein liquid chromatography (FPLC). Eluent: 0.15 mol/L NaCl in 0.05 mol/L phosphate buffer (pH 7.5). Flow rate: 0.5 mL/min. Fraction S2: purified polysaccharide (HRP).

CHRP was dissolved in deionized water and deproteinized in n-butanol/chloroform (v/v; 4:1) for 4 h. After stratification, upper fraction was collected and dialyzed thoroughly against phosphate buffer (0.05 mol/L, pH 7.4) at room temperature. Dialyzed CHRP solution was subjected to DEAE-Sepharose chromatography, eluted with phosphate buffer, and unbound fraction (DHRP) was collected. DHRP was dialyzed against acetate buffer (0.05 mol/L, pH 4.6) at room temperature, subjected to CM-Sepharose chromatography, eluted with acetate buffer, and unbound fraction (CDHRP) was collected. CDHRP was subjected to gel filtration (Superdex 75 16/600 column, GE Healthcare, United States) using an AKTA purifier (Amersham Biosciences, Sweden), and eluted with 0.15 mol/L NaCl in 0.05 mol/L phosphate buffer (pH 7.5) at flow rate 0.5 mL/min. Fractions were collected with an automated fraction collector, and the peak with highest polysaccharide content (S2) was collected. Fraction S2 was dialyzed in deionized water for 48 h with a dialysis bag (MW cutoff 3.5 kDa), freeze-dried, and termed HRP (Figure 2).

Polysaccharide content was determined by DuBois’ method (20). 1.5 mL polysaccharide solution was mixed with 5 mL sulfuric acid, and the mixture was kept in boiling water bath for 20 min and then cooled to room temperature. Absorption value was determined by spectrophotometry at wavelength 490 nm, and carbohydrate concentration was estimated based on a standard curve.

### Molecular Weights and Monosaccharide Composition of *Heimioporus retisporus* Polysaccharide

Weight-average (*M*_w_) and number-average (*M*_n_) molecular weights of HRP were determined by gel permeation chromatography (GPC) using an Agilent 1200 HPLC system equipped with PL aquagel-OH 50 column (7.7 × 300 mm) and differential refractive index detector. Samples (5.0 mg) were dissolved in 1.0 mL phosphate buffer (0.2 mol/L, pH 7.5) containing 1.0 mL NaCl (0.02 mol/L), and filtered through a membrane (pore size 0.45 μm). For each run, 20 μL solution (0.1 mg HRP) was injected and eluted with phosphate buffer (flow rate 0.5 mL/min, 30°C). *M*_w_ and *M*_n_ values were estimated using a calibration equation based on PL pullulan polysaccharide standards.

Monosaccharide composition of HRP was analyzed by high-performance anion exchange chromatography (HPAEC) coupled with pulsed amperometric detector (PAD). Neutral sugars and uronic acids were released by hydrolysis (10% H_2_SO_4_, 2.5 h, 105°C). Acid hydrolysates of HRP were diluted and analyzed using HPAEC system (Dionex ISC 3000; United States) with PAD, AS50 autosampler, CarboPac PA20 column (4 × 250 mm, Dionex), and PA-20 guard column (3 × 30 mm). Standard solutions of L-arabinose, D-glucose, D-xylose, D-glucose, D-mannose, D-galactose, glucuronic acid, and galacturonic acid were used for calibration.

### Fourier Transform Infrared Spectroscopy

Fourier transform infrared spectroscopy was performed using Optik GmbH Tensor II system (Bruker, Germany). Spectra were recorded from 4,000 to 400 cm^–1^, with resolution 4 cm^–1^ and maximal source aperture (21).

### Nuclear Magnetic Resonance Spectroscopy

∼40 mg HRP was dissolved in 0.55 mL chloroform-d (CDCl_3_), and solution-state ^1^H and ^13^C nuclear magnetic resonance (NMR) spectroscopy (Bruker system) were performed with parameters: spectral width 1,800 Hz for ^1^H dimension and 10,000 Hz for ^13^C dimension; delay between transients 2.6 s; delay for polarization transfer corresponding to estimated average ^1^H-^13^C coupling constant 150 Hz. Data were processed using Bruker Topspin-NMR software program (22).

### Thermogravimetric Analysis

Thermogravimetric analysis (TGA) and derivative thermogravimetry (DTG) were performed using simultaneous thermal analyzer (model STA449F3; Netzsch; Germany). ∼8 mg lyophilized HRP powder was placed in a platinum crucible under nitrogen atmosphere, and heated at rate 10°C/min in temperature range of 30–800°C (23). Data were analyzed using software program Origin 8.0.2.8.

### Scanning Electron Microscopy

Dried HRP samples were gold-coated by sputter-coater (model IB-3, EiKo, Japan), and morphological features were observed by scanning electron microscopy (model S-3400N, Hitachi, Japan) (accelerating voltage 10.0 kV; magnifications ×100, ×500, ×1,000, ×2,000; high vacuum conditions) as described previously (24).

### Animal Model and Drug Administration

Male SPF Balb/c mice (weight 20 ± 2 g) from Charles River (Beijing) were maintained in the Experimental Animal Public Service Platform at China Agricultural University (25 ± 2°C, humidity 50 ± 10%, 12 h light/12 h dark cycle), and fed normal chow diet *ad lib*. After 1 wk acclimination period, mice were i.p. injected with 1% STZ (40 mg/kg) for 5 days (25), and fasting blood glucose (FBG) was measured 24 h after the last injection. A successful model was defined as mice with FBG ≥ 11.0 mmol/L, stable for 1 week (26).

Group Blank consisted of five untreated normal mice. 25 diabetic mice were assigned randomly to one control and four experimental groups (intragastric administration; 4-wk feeding period) as follows:

Group Blank: blank, deionized water.

Group CK: deionized water; control.

Group Met: metformin (40 mg/kg); positive control.

Group HRP-20: HRP 20 mg/kg.

Group HRP-40: HRP 40 mg/kg.

Group HRP-80: HRP 80 mg/kg.

Body weight and FBG data were collected for the four experimental groups. At the end of experimental period, mice were sacrificed (cervical dislocation), heart, liver, spleen, and kidney were removed, connective tissue was cleaned and washed with saline to remove blood, and collected organs were weighed for calculation of organ indices, immediately frozen in liquid nitrogen, and stored at -80°C for further analysis. All experiments were approved by the Institutional Ethics Committee of China Agricultural University, and performed in accordance with International Standards and Ethical Guidelines for Animal Welfare.

### Fasting Blood Glucose Measurement

Mice were fasted for 8 h, blood was extracted from tail vein, and the FBG was measured using express glucose meter (On Call).

### Visceral Organ Indices

For each of various visceral organs, an index was calculated by the formula:


$$\begin{array}{c} \mathrm{Visceral}\mathrm{organ}\mathrm{index}\\ =\left(\text{v}\mathrm{isceral}\mathrm{organ}\mathrm{weight}\right)/\left(\text{b}\mathrm{ody}\mathrm{weight}\right)\times 100\% \end{array}$$

### Inflammatory Cytokine mRNA Levels in Heart Tissue

Total RNA extraction from heart tissue was performed using TRIzol reagent. First-strand cDNA synthesis was performed using a commercial reverse transcription kit as per manufacturer’s instructions. Primer sequences used were as follows:

**Table:** 

β-actin	forward primer	5′-AACACCCCAGCCATGTACG-3′
	reverse primer	5′-ATGTCA CGCACGATTTCCC-3′
IL-6	forward primer	5′-TGCTGGTGACAACCACGGCC-3′
	reverse primer	5′ -GTACTCCAGAAGACCAGAGG-3′
INF-α	forward primer	5′-ATGGCCTCCCTCTCATCAGT-3′
	reverse primer	5′-ATAGCAAATCGGCTGACGGT-3′

PCR procedure was: initial denaturation at 94°C for 4 min, 30 cycles of denaturation at 94°C for 30 s, annealing at 60°C (β-actin) or 62°C (IL-6, TNF-α) for 30 s, extension at 72°C for 30 s, final extension at 72°C for 10 min. Amplification products were confirmed by electrophoresis (1.0% agarose gels) and visualized by Gel Red staining (27).

### Statistical Analysis

Results were expressed as mean ± SE, and data were analyzed using software program SPSS 20.0 (IBM). Means were compared by one-way analysis of variance (ANOVA), with *p* < 0.05 as criterion for significant difference.

## Results

### *Heimioporus retisporus* Polysaccharide Molecular Weights and Monosaccharide Components

*M*_w_ and *M*_n_ of HRP were, respectively, 1,949 and 873.34 kDa, and polydispersity index (PDI, calculated as *M*_w_/*M*_n_) was 2.232. PDI reflects distribution of molecular weight in each polymer sample, and the low value indicates that chain lengths of HRP vary over a relatively narrow range of molecular weights.

Monosaccharide composition of HRP, determined by HPAEC/PAD analysis, is summarized in Table 1. The major component was glucose (49.0%), followed by mannose, galactose, glucuronic acid, and xylose (percentages ranging from 17.8 to 8.6%). Arabinose was a minor component (0.7%).

**TABLE 1:** Monosaccharide composition of *Heimioporus retisporus* Polysaccharide (HRP).

Monosaccharide:	Glucose	Mannose	Galactose	Glucuronic acid	Xylose	Arabinose
Molar ratio (%):	49.00	17.78	12.93	10.99	8.59	0.71

### Fourier Transform Infrared Spectroscopy Analysis

**FIGURE 3:**
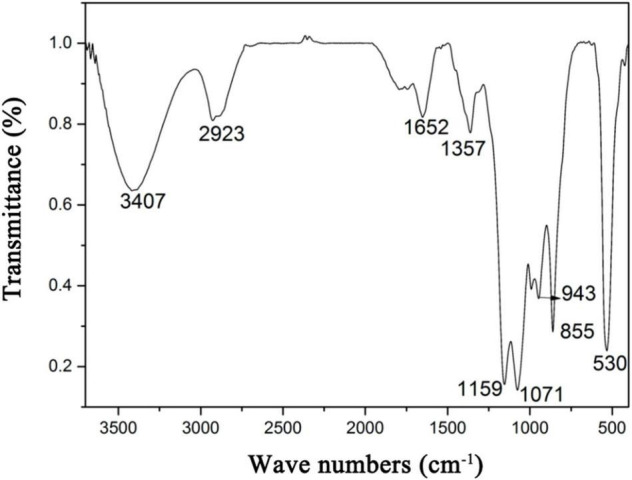
Fourier transform infrared spectroscopy (FT-IR) spectrum of *Heimioporus retisporus* Polysaccharide (HRP).

In the FT-IR spectrum of HRP (Figure 3), the absorption bands at 3,407 and 2,923 cm^–1^ represent stretching vibrations of O-H and C-H groups of the sugar ring (28). The 1,652 cm^–1^ band reflects C=O linkage (29), weak symmetric stretching band near 1,357 cm^–1^ corresponds to carboxylate groups (30), 1,159 cm^–1^ band reflects stretching of α-(1,4) glycosidic linkages (31), 1,071 cm^–1^ band indicates that HRP sugar rings are pyranose rings (32), 855 cm^–1^ peak represents α-glycosidic bonds (33), 943 cm^–1^ band represents β-glycosidic bonds (34), and 530 cm^–1^ band reflects in-plane C=O bending (35).

### Nuclear Magnetic Resonance Analyses

**FIGURE 4:**
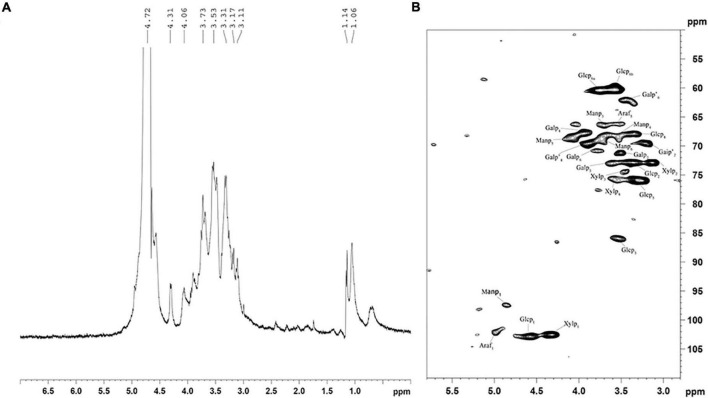
^1^H **(A)** and HSQC **(B)** spectra of *Heimioporus retisporus* Polysaccharide (HRP). Glcp: →3)-β-D-Glcp-(1→; Manp: →6)-β-D-Manp-(1→; Galp: →6)-β-D-Galp-(1→; Galp’: →4)-β-D-Galp-(1→; Xylp: →4)-β-D-Xylan-(1→; Araf: →5)-α-L-Araf-(1→.

Structural features of HRP were elucidated by measuring 1-D NMR (^1^H) and 2-D NMR (heteronuclear single quantum coherence; HSQC) spectra. In the ^1^H NMR spectrum (Figure 4A), H-1 signals representing six residues were seen at 3.11, 3.17, 3.31, 3.51, 3.73, and 4.06 ppm. The first four (strong signals) indicate presence of β-D-glucose (36), and the latter two reflect β-D-mannose configured residues (37).

In the HSQC spectrum (Figure 4B), six cross-peaks (4.48/102.9, 3.36/72.8, 3.51/85.8, 3.34/68.0, 3.38/76.0, and 3.64, 3.58/60.4 ppm) were assigned, respectively, to H-1/C-1, H-2/C-2, H-3/C-3, H-4/C-4, H-5/C-5, and H-6(a), H-6(b)/C-6 of →3)-β-D-Glcp-(1→residues. ^1^H/^13^C chemical shifts at 3.51/71.2, 73.1/3.62, 68.8/3.95, and 3.78/70.9 ppm were assigned to H-2/C-2, H-3/C-3, H-4/C-4, and H-6/C-6 of →6)-β-D-Galp-(1→residues, and cross-peaks at 3.43/69.8, 3.88/69.3, and 3.41/62.4 were assigned to H-2/C-2, H-4/C-4, and H-6/C-6 of →3)-β-D-Galp-(1→residues. Cross-peaks at 4.22/102.2, 3.13/73, 3.46/74.2, and 3.62/75.8 ppm were assigned to H-1/C-1, H-2/C-2, H-3/C-3, H-4/C-4 of →4)-β-D-xylan-(1→, and those at 4.86/97.2, 3.71/66.2, 3.58/68.0, 4.08/68.2, and 3.72/68.2 were assigned to H-1/C-1, H-3/C-3, H-4/C-4, H-5/C-5, and H-6/C-6 of →6)-β-D-Manp-(1→residues. Cross-peaks at 4.99/102.1 and 3.50/66.30 were assigned to →5)-α-L-Araf-(1→residues (36, 38–40). These findings are consistent with those for monosaccharide composition and ^1^H spectra.

### Thermal Analysis

**FIGURE 5:**
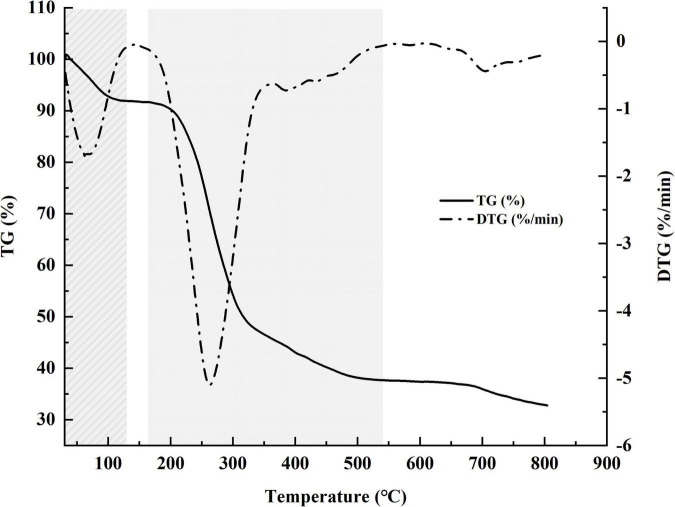
Weight loss (TG) percentage and derivative thermogravimetric (DTG) curves for *Heimioporus retisporus* Polysaccharide (HRP).

Weight loss (TG) and DTG curves of samples are shown in Figure 5. The TG curve shows two stages (30–130 and 165–540°C) of weight loss. The first stage (∼9%), resulting from vaporization and removal of bound water in HRP, reflects characteristic moisture sorption based on abundance of hydroxyl radical. The second (degrading) stage, involving alteration of functional groups and depolymerization of structure, resulted in substantial loss (∼54%) of sample weight. The two curves indicate onset degradation temperature (T_0_) = 200°C and maximum degradation temperature (T_max_) = 263°C.

### Microstructural Analysis

**FIGURE 6:**
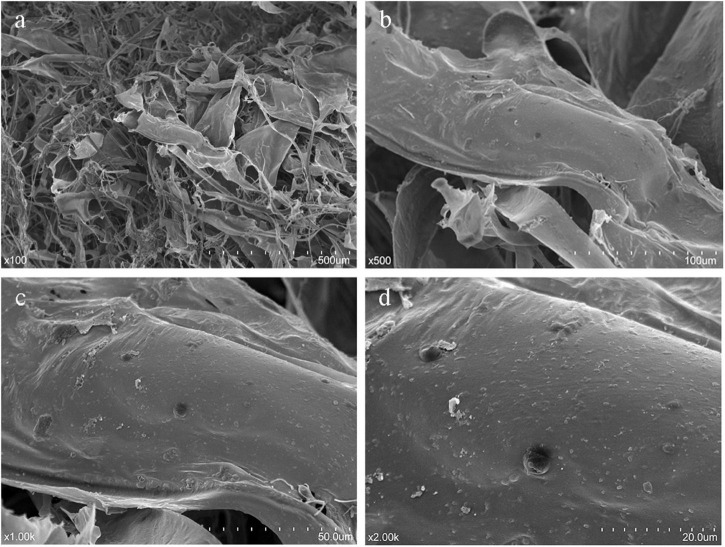
Scanning electron microscopy (SEM) imaging of *Heimioporus retisporus* Polysaccharide (HRP). **(a–d)** magnifications ×100, ×500, ×1,000, ×2,000.

Analysis of surface morphology by SEM is a qualitative tool to characterize polysaccharides. SEM images of HRP (magnifications ×100, ×500, ×1,000, ×2,000) (Figure 6) demonstrate an entanglement structure with irregular sheets and coils. The tangled structure reflects the complex nature of HRP (41). The apparent pores may be artifacts of the freeze-drying process.

### Antidiabetic Effect

**FIGURE 7:**
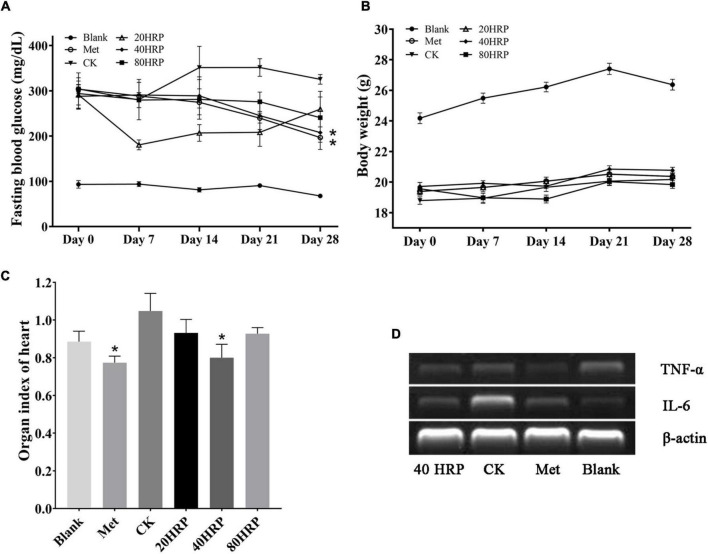
Antidiabetic effect of *Heimioporus retisporus* Polysaccharide (HRP). **(A)** Fasting blood glucose (FBG) level. **(B)** Weight. **(C)** Visceral organ index of heart. **(D)** RT-PCR of TNF-α and IL-6. Values are expressed as mean ± SE. *p < 0.05 for comparison with CK group.

Figure 7 illustrates *in vivo* antidiabetic effect of HRP. FBG levels on day 28 were significantly lower for Groups HRP-40 and Met than for Group CK. Group HRP-20 had a striking reduction in FBG level between days 0 and 7. No such reduction was observed for Group HRP-80, suggesting that the effect of HRP was not dose-dependent (Figure 7A). No notable effects on body weight were observed for the experimental groups (Figure 7B).

Visceral organ indices did not differ significantly for Group CK vs. the three HRP groups, except for heart (data not shown). Heart indices are shown in Figure 7C. Heart index for Group CK was higher than those of all the other groups, and the difference was significant for Groups Met and HRP-40. RT-PCR assays for inflammatory cytokines IL-6 and TNF-α showed that IL-6 expression levels in heart tissue were higher for Group CK than for Group Met or the HRP groups, while TNF-α expression levels were similar for all groups. These findings are consistent with the high heart index for Group CK (Figure 7D).

## Discussion

Numerous polysaccharides from medicinal mushroom species have been shown to display hypoglycemic activity, but reported structures and activities of such polysaccharides are highly variable depending on extraction and purification methods (42). We used hot water extraction to purify and characterize a novel neutral polysaccharide from the mushroom *H. retisporus* (termed HRP), and demonstrated strong hypoglycemic activity of HRP in an STZ-induced diabetic mouse model.

HRP is composed of glucose (the predominant component), mannose, galactose, glucuronic acid, xylose, and arabinose, in molar ratio 49.00: 17.78: 12.93: 10.99: 8.59: 0.71% (Table 1). The monosaccharides glucose, galactose and mannose are in β-D conformations. The polysaccharides GLP-1 and GLP-2 from *Ganoderma lucidum* are composed of mannose, glucose, galactose, and fucose in respective molar ratios 4.9: 63.5: 26.2: 5.4% and 1.6: 90.6: 7.8: 0% (43). The polysaccharide CFP from *Pleurotus citrinopileatus* is composed of galactose, glucose, glucuronic acid, and glucuronic acid in molar ratio 20.53: 28.75: 5.55: 45.17% (44). Most medicinal fungal polysaccharides have glucose as the major component (45), but there is great variability in identities and proportions of other components.

Intragastric administration of 40 mg/kg HRP in an STZ-induced diabetic mouse model caused significant reduction of blood glucose level, but had no notable effect on body weight. HRP was found to decrease visceral organ index for heart, and we therefore used RT-PCR assay to evaluate expression levels of inflammatory cytokines IL-6 and TNF-α in heart. IL-6 expression level was reduced by HRP treatment. Previous investigations of elevated tissue concentrations of inflammatory cytokines in mouse diabetes models indicate that inflammatory processes promote development of diabetic cardiomyopathy. For example, intramyocardial inflammation (including increased expression of IL-6 or TNF-α) contributed to diabetic cardiomyopathy (3). Activated macrophages enhance the production of IL-6, but excessive activation of macrophages can cause damage to living organisms (46). HRP may protect the heart by preventing excessive activation of macrophages.

Many recent studies have revealed hypoglycemic effects of certain polysaccharides (45, 47, 48). One example is a polysaccharide (SERP1) from the herb *Sarcandra glabra* (family Chloranthaceae) composed of 1,4-linked α-D-galacturonic acid, methyl esterified 1,4-linked α-D-galacturonic acid, 1,4-linked α-D-glucuronic acid, 1,5-linked α-L-arabinose, 1,3-linked β-D-galactose, 1,4-linked α-D-glucose, 1,4,6-linked β-D-glucose, 1,6-linked β-D-glucose, and 1,2-linked rhamnose (49). HRP in this study was composed of 1,3-linked β-D-glucose, 1,6-linked β-D-mannose, 1,6-linked β-D-galactose, 1,4-linked β-D-galactose, 1,4-linked β-D-xylose, and 1,5-linked α-L-arabinose. HRP and SERP1 thus have similar monosaccharide compositions, but different linkages. More generally, there are numerous naturally occurring polysaccharides that display hypoglycemic activity, but none of them have the same compositions, linkages, or conformations (34, 49–55). There is no direct evidence that polysaccharide components control hypoglycemic activity based on ratios of specific monosaccharides. On the other hand, several studies suggest that mannogalactoglucan domain plays a role in suppressing hyperglycemia, consistent with our findings (45, 56, 57). Yang et al. (45) analyzed hypoglycemic activity of 18 polysaccharides extracted from fruiting bodies of various mushroom species. In a db/db mouse model, neutral polysaccharide AAMP-N, which has a large mannogalactoglucan domain, strongly enhanced insulin sensitivity *in vitro*, reduced FBG, and modulated lipid metabolism. Future studies by our group and others will elucidate the link between structural characteristics of polysaccharides and their hypoglycemic activities.

Therefore, we characterized HRP, a water-soluble neutral polysaccharide extracted from *H. retisporus*, as a heteropolysaccharide composed of 1,3-linked β-D-glucose, 1,6-linked β-D-mannose, 1,6-linked β-D-galactose, 1,4-linked β-D-galactose, 1,4-linked β-D-xylose, and 1,5-linked α-L-arabinose. In an STZ-induced diabetic mouse model, HRP significantly reduced blood glucose level and heart visceral organ index by downregulating IL-6 expression. HRP has strong potential for application as a hypoglycemic, cardioprotective dietary supplement in diabetes treatment.

## Compliance With Ethical Standards

Consent for publication: All authors listed on this manuscript have read and agreed to the publication of this research.

## Data Availability Statement

The original contributions presented in the study are included in the article/supplementary material, further inquiries can be directed to the corresponding authors.

## Ethics Statement

The animal study was reviewed and approved by the Institutional Ethics Committee of the China Agricultural University (No: CAU20180420-5).

## Author Contributions

QL conceived and designed the study. QL, XF, PW, YL, and ZZ performed the purification, characterization, bioactivity assay, and analyzed data. CY, GT, and QL identified and collected *Heimioporus retisporus* fruiting bodies. QL, XF, and PW wrote the manuscript. All authors read and approved the manuscript in its finalized form.

## Conflict of Interest

The authors declare that the research was conducted in the absence of any commercial or financial relationships that could be construed as a potential conflict of interest.

## Publisher’s Note

All claims expressed in this article are solely those of the authors and do not necessarily represent those of their affiliated organizations, or those of the publisher, the editors and the reviewers. Any product that may be evaluated in this article, or claim that may be made by its manufacturer, is not guaranteed or endorsed by the publisher.
